# Cellulose Dissolution in Mixtures of Ionic Liquids and Dimethyl Sulfoxide: A Quantitative Assessment of the Relative Importance of Temperature and Composition of the Binary Solvent

**DOI:** 10.3390/molecules25245975

**Published:** 2020-12-17

**Authors:** Marcella T. Dignani, Thaís A. Bioni, Thiago R. L. C. Paixão, Omar A. El Seoud

**Affiliations:** Institute of Chemistry, The University of São Paulo, 748 Prof. Lineu Prestes Av., São Paulo 05508-000, Brazil; marcelladignani2@gmail.com (M.T.D.); tha.bioni@hotmail.com (T.A.B.); trlcp@iq.usp.br (T.R.L.C.P.)

**Keywords:** assessment of cellulose dissolution, ionic liquids, dimethyl sulfoxide, chemometrics, medium empirical polarity

## Abstract

We studied the dissolution of microcrystalline cellulose (MCC) in binary mixtures of dimethyl sulfoxide (DMSO) and the ionic liquids: allylbenzyldimethylammonium acetate; 1-(2-methoxyethyl)-3-methylimidazolium acetate; 1,8-diazabicyclo [5.4.0]undec-7-ene-8-ium acetate; tetramethylguanidinium acetate. Using chemometrics, we determined the dependence of the mass fraction (in %) of dissolved cellulose (MCC-m%) on the temperature, T = 40, 60, and 80 °C, and the mole fraction of DMSO, χ**_DMSO_** = 0.4, 0.6, and 0.8. We derived equations that *quantified* the dependence of MCC-m% on T and χ**_DMSO_**. Cellulose dissolution increased as a function of increasing both variables; the contribution of χ**_DMSO_** was larger than that of T in some cases. Solvent empirical polarity was *qualitatively* employed to rationalize the cellulose dissolution efficiency of the solvent. Using the solvatochromic probe 2,6-dichloro-4-(2,4,6-triphenylpyridinium-1-yl)phenolate (WB), we calculated the empirical polarity *E**_T_***(WB) of cellobiose (a model for MCC) in ionic liquid (IL)–DMSO mixtures. The *E**_T_***(WB) correlated perfectly with T (fixed χ**_DMSO_**) and with χ**_DMSO_** (fixed T). These results show that there is ground for using medium empirical polarity to assess cellulose dissolution efficiency. We calculated values of MCC-m% under conditions other than those employed to generate the statistical model and determined the corresponding MCC-m% experimentally. The excellent agreement between both values shows the robustness of the statistical model and the usefulness of our approach to predict cellulose dissolution, thus saving time, labor, and material.

## 1. Introduction

The need to increase the use of cellulose (Cel) from sources other than cotton, in particular from wood, has resulted in increased interest in optimizing the efficiency of solvents for the *physical dissolution* of Cel, i.e., without the formation of covalent bonds [1,2,3,4].

Ionic solvents, in particular ionic liquids (ILs) based on imidazole, quaternary ammonium- and phosphonium electrolytes, and salts of superbases (e.g., 1,5-diazabicyclo [4.3.0]non-5-ene (DBN) and 1,8-diazabicyclo[5.4.0]undec-7-ene (DBU)), these ILs have been employed both pure and as binary mixtures with dipolar aprotic molecular solvents [5]. A central issue in these studies was to maximize the concentration of dissolved Cel. Binary mixtures (BMs) of ILs with molecular solvents are frequently used as cellulose solvents. In addition to a better mass/heat transfer due to the lower viscosity, many dissolve more Cel than their precursor ILs [6,7]. Because of its efficiency in Cel swelling [8] and high dipole moment and relative permittivity, dimethyl sulfoxide (DMSO) is most extensively employed as a co-solvent for ILs.

For simplicity, we used the following definitions/acronyms in the subsequent discussion: microcrystalline cellulose (MCC) to represent Cel, mass fraction of dissolved MCC (MCC-m%; see Equation (1)), and mole fraction of dissolved MCC (MCC-χ; see Equation (2); AGU = anhydroglucose unit). For brevity, we limited our discussion to MCC-m%. As we will show below, the use of MCC-m% or MCC-χ leads to identical conclusions.

MCC-m% = [mass of dissolved MCC/(mass of dissolved MCC + mass of ionic liquid + mass of DMSO)] × 100(1)

MCC-χ = [number of moles of dissolved AGU/(number of moles of dissolved AGU + number of moles of IL + number of moles of DMSO)](2)

The usual procedure for optimizing the dissolution of Cel in a solvent is to change one experimental variable (or factor) at a time, e.g., temperature or composition of the BM. If needed, the first factor is fixed at its value that resulted in the maximum MCC-m%, and the other factor (e.g., BM composition) is then varied until a *new* maximum MCC-m% is reached [7,9,10,11,12,13]. Although useful, this approach does not guarantee *a real* MCC-m% maximum *for both variables*. Equally important, however, this one-at-a-time approach gives no indication about the relative importance to Cel dissolution of the experimental variables. Therefore, chemometrics should be used, where both factors are varied *simultaneously in a random manner* [14].

**Figure 1:**
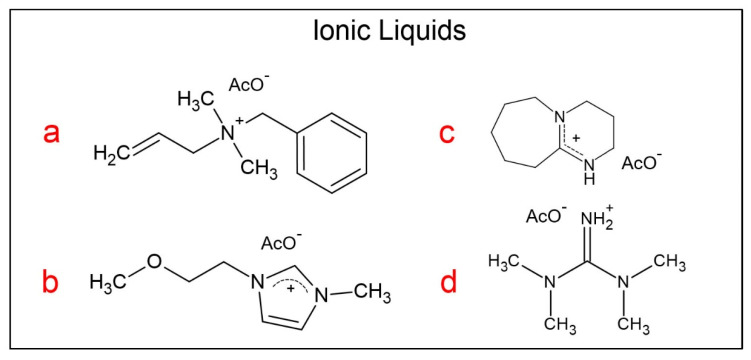
Molecular structures of the ionic liquids (ILs) employed, including: (**a**) allylbenzyldimethylammonium acetate (AlBzMe_2_NAcO); (**b**) 1-(2-methoxyethyl)-3-methylimidazolium acetate (C_3_OMeImAcO); (**c**) 1,8-diazabicyclo[5.4.0]undec-7-ene-8-ium acetate (DBUHAcO); (**d**) tetramethylguanidinium acetate (TMGHAcO).

Using a recently constructed equipment that ensures reproducible Cel dissolution data and a recommended dissolution protocol [15], we employed chemometrics to optimize MCC-m% for biopolymer dissolution in binary mixtures of dimethyl sulfoxide (DMSO) with the following ionic liquids: allylbenzyldimethylammonium acetate (AlBzMe_2_NAcO); 1-(2-methoxyethyl)-3-methylimidazolium acetate (C_3_OMeImAcO), 1,8-diazabicyclo[5.4.0]undec-7-ene-8-ium acetate (DBUHAcO), and tetramethylguanidinium acetate (TMGHAcO) (Figure 1).

The experimental variables studied were the dissolution temperature (T = 40, 60, and 80 °C) and the DMSO mole fraction (χ**_DMSO_** = 0.4, 0.6, and 0.8). We obtained equations that correlate MCC-m% with T and χ**_DMSO_**. These indicated that increasing the values of both factors favor MCC dissolution; the latter variable is more important in some cases.

Many authors used solvent empirical polarity (vide infra) to *qualitatively* rationalize the effects of pure solvents and BMs on Cel dissolution [6,16,17]. Therefore, we used the solvatochromic probe 2,6-dichloro-4-(2,4,6-triphenylpyridinium-1-yl)phenolate (WB) and cellobiose as a model for MCC and calculated the empirical polarity, *E**_T_***(WB), of the disaccharide/IL–DMSO solutions. We found perfect second-order polynomial correlations between *E**_T_***(WB) and T (at fixed χ**_DMSO_**) and between *E**_T_***(WB) and χ**_DMSO_** (at fixed T). Thus, there is *theoretical and experimental* ground for using the empirical polarity of a medium to explain its efficiency as a Cel solvent.

The generated statistical equations reproduce satisfactorily MCC dissolution data at T and χ**_DMSO_** values *other than those employed to create the statistical model*. This agreement shows the robustness of the chemometric approach used. Equally important, however, is that the model permits the prediction of biopolymer dissolution, saving time, labor, and material.

## 2. Materials and Methods

### 2.1. Materials

The reagents used were purchased from Sigma–Aldrich or Merck. The MCC, Avicel PH 101 (viscosity-average degree of polymerization = 155 ± 3, Ref. [18] was purchased from FMC. Before use, the MCC was dried for 2 h at 70 °C under reduced pressure.

### 2.2. Synthesis of the Ionic Liquids

The DBUHAcO and TMGHAcO were synthesized according to the literature, [17] by mixing equimolar quantities of *standardized solutions* of the superbase (DBU and/or TMG) with acetic acid in acetonitrile (MeCN), followed by stirring for 2 h at room temperature and removal of MeCN.

The AlBzMe_2_NAcO and C_3_OMeImAcO were synthesized as given elsewhere [19,20,21]. *N*-Benzyl-*N,N*-dimethylamine was reacted with allyl bromide in MeCN. After solvent removal, the produced allylbenzyldimethylammonium bromide was purified by suspension in cold ethyl acetate (3 times; vigorous agitation), followed by removal of the latter. The obtained bromide was transformed into the corresponding acetate by ion exchange on a macroporous resin in the acetate form (Amberlite IRN 78, 1.20 equivalent acetate ions/L resin), using methanol as eluent, followed by removal of the latter. *N*-Methylimidazole was reacted with 1-chloro-2-methoxyethane in MeCN under pressure (10 atm, 6 h, 85 °C), followed by removal of MeCN and purification by agitation with ethyl acetate, vide supra. The produced 1-(2-methoxyethyl)-3-methylimidazolium chloride was transformed into C_3_OMeImAcO using ion exchange, as given for AlBzMe_2_NAcO.

The synthesized ILs were dried under reduced pressure, in the presence of P_4_O_10_. We analyzed the purity of these products by ^1^H NMR spectroscopy (Varian Inova model YH300 spectrometer; 300 MHz for ^1^H; CDCl_3_ solvent). Each IL gave the expected spectrum (See Supplementary Materials Figure S1).

### 2.3. Determination of the Concentration Dissolved Cellulose in IL–DMSO

**Figure 2:**
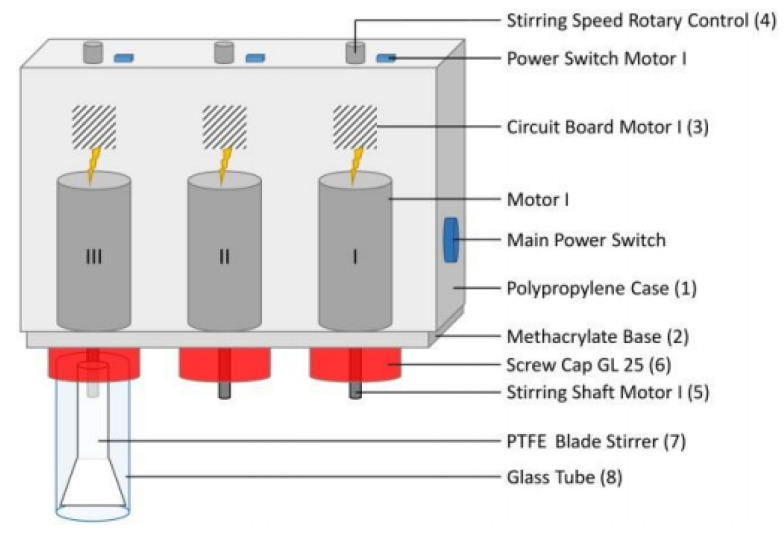
Schematic representation of the equipment employed for the quantification of cellulose dissolution. The equipment parts included: mini-motors with controlled rotation speed connected to a PTFE blade through a screw cap. Dissolution occurs in a glass tube equipped with a thread. The glass tubes are immersed in a controlled temperature water bath [15].

**Figure 3:**
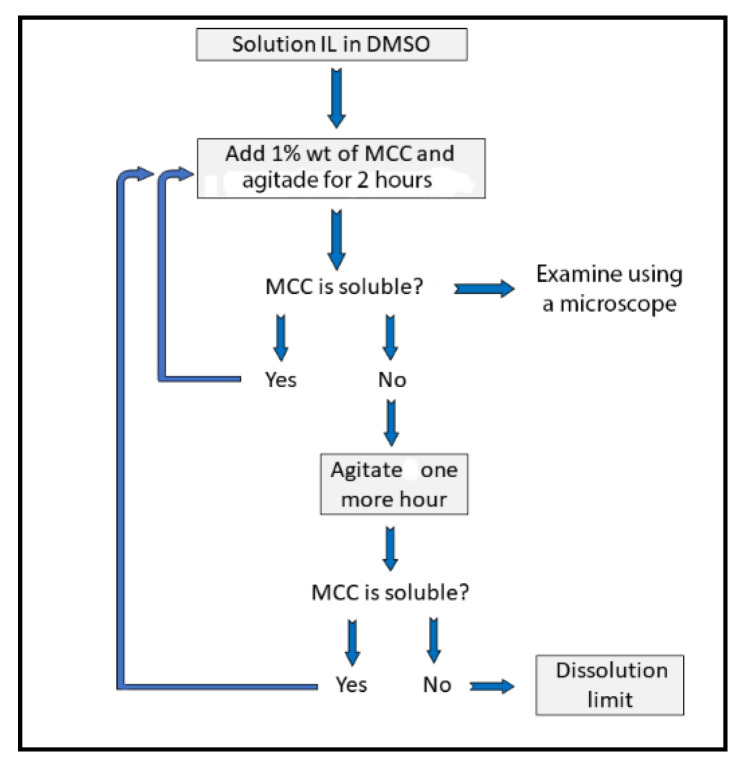
Flow chart for the steps of the cellulose dissolution experiment. Microcrystalline cellulose, (MCC); dimethyl sulfoxide, (DMSO).

**Figure 4:**
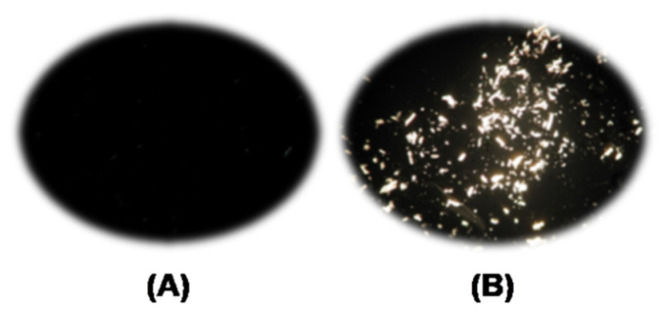
View of MCC dissolution using a polarized-light microscope: (**A**) complete and (**B**) partial cellulose dissolution.

Figure 2 shows the equipment employed for the MCC dissolution; its parts are explained in the corresponding legend. The dissolution experiments were carried out as follows (see the flow chart of Figure 3): Known masses of the IL–DMSO BM with the desired χ**_DMSO_** (approximately 3 g) and MCC (approximately 10–30 mg) were quickly weighted into a threaded glass tube. The latter was attached to the mechanical stirrer and then introduced into a thermostated polymethacrylate water bath already set at the desired temperature (Isotemp 730 heating unit, Fisher Scientific; Aumax model N1540 digital thermometer, bath temperature constant within ±0.2 °C). The suspension was agitated at a constant speed (300 rpm, measured with a digital laser tachometer, model 2234C^+^, Signsmeter). The MCC dissolution was judged visually, *without opening the glass tube*, under 12× magnifying glass provided with an LED light. The final decision (on MCC dissolution) was reached with the aid of a microscope (Nikon, Tokyo, Japan, Eclipse 2000 microscope with cross polarization). Complete cellulose dissolution was evidenced by observing a clear (i.e., dark) view, see Figure 4A. If complete dissolution was not observed after 2 h of agitation (Figure 4B), the suspension was agitated further for an additional hour. We considered that MCC maximum dissolution was reached when the biopolymer remained undissolved after 3 h *from the last solid addition*.

### 2.4. Calculation of the Empirical Solution Polarity, E_T_(WB)

We used Equation (3) to calculate the solution empirical polarity using WB as a solvatochromic probe [22]:

*E**_T_***(WB), kcal/mole = 28591.5/λ_max_, nm (3)

where λ_max_ is the wavelength of the solvatochromic peak, and *E**_T_***(WB) is the corresponding empirical polarity. Solutions of MCC in IL–DMSO scatter visible light, especially at high biopolymer concentrations. Therefore, we used cellobiose (CB) as a model for Cel [19,23,24] and prepared solutions that contained CB concentration (CB-m%) *equivalent* to (MCC-m%). We pipetted 50 μL of the WB stock solution in acetone (1 × 10^−^^3^ mol L^−1^) into glass vials and removed the solvent under reduced pressure in the presence of P_4_O_10_. To each vial we added 1 mL of the appropriate CB-m%/IL–DMSO solution, dissolved the solid probe (vortex agitation), and transferred the resulting *clear solution* to a 1 cm path length semi-micro quartz cell with a PTFE stopper. We recorded each spectrum twice at a resolution of 0.2 nm, at 30, 40, 50, and 60 °C using a Shimadzu UV-2550 UV-Vis spectrophotometer provided with a digital thermometer (model 4000A, Yellow Springs Instruments, Yero Springs, OH, USA). We calculated the value of λ_max_ from the first derivative of the spectrum. The value of *E_T_*(WB) at 80 °C was calculated by extrapolation of the *E_T_*(WB) versus T curve.

### 2.5. Statistical Design of the Cellulose Dissolution Experiment 

Design of experiments (DOE) [25,26] is a systematic method to determine the relationship between experimental variables or factors affecting the output of a process. This method is used to find cause-and-effect relationships [14]. To perform DOE, we used the Statistica Software (version 13.0, Dell, Austin, TX, USA). The order of design points was randomized to reduce the effect of unpredicted variables (vide infra); the total number of experiments for each MCC/IL–DMSO was 16. A quadratic model was fitted to the dissolution data, see Section 3 (Results and Discussion). Response surfaces (vide infra) were generated by the response surface methodology (RSM) as implemented in the Statistica software.

## 3. Results and Discussion

### Design of the Cellulose Dissolution Experiments

As shown in Section 2, we used one cellulose sample (MCC) and carried out the dissolution experiment at a fixed stirring rate. Therefore, the only experimental variables were T and χ**_DMSO_**. Each variable had three values or *levels*. According to DOE, the minimum number of experiments is 9 (i.e., equal to the number of levels^(number of variables)^ = 3^2^). In order to increase the robustness of the model, we repeated the central point (T = 60 °C and χ**_DMSO_** = 0.6) three more times and repeated the minimum and maximum levels (T = 40, 80 °C; χ**_DMSO_** = 0.4, 0.8) one more time, *giving 16 experiments* for each IL–DMSO BM (9 + 3 + (2 × 2)).

It is customary to denote the low, intermediate, and high values of factor levels by −1, 0, and +1, respectively. For example, we designated 40, 60, and 80 °C by −1, 0, and 1. Table S1 shows the randomized order of experiments using this designation as generated by the software employed. Table 1 shows the experimentally determined dependence of MCC-m% and MCC-χ on T and χ**_DMSO_**. In Table 1, we list in footer (^d^) the empirical polarity *E**_T_***(WB) of the mixtures (CB + IL + DMSO) at different temperatures and DMSO mole fractions.

As there were two experimental variables, we expected that a quadratic model should fit our data [14] as given by Equation (4) for the present case:

MCC-m% or χ**_MCC_** = Constant + a_1_ T + a_2_ χ**_DMSO_** + a_3_ T^2^ + a_4_ (χ**_DMSO_**)^2^ + a_5_ (Tx χ**_DMSO_**) (4)

where a_1_, a_2_, etc., are regression coefficients. Note that whereas the terms in T and χ**_DMSO_** are related *directly* to the variables studied, the quadratic and “cross” terms (i.e., T^2^, (DMSO)^2^, and Tx χ**_DMSO_**) are required for a better statistical fit of the model to the data. In other words, the term a_4_ (χ**_DMSO_**)^2^ does not necessarily mean that Cel dissolution occurs by the dimer of DMSO.

**Figure 5:**
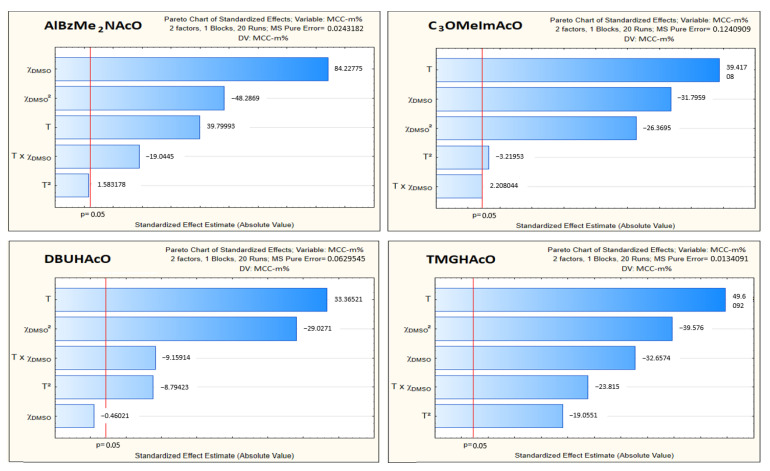
Pareto charts for the relative importance to cellulose dissolution of the temperature (T) and the mole fraction of DMSO (χ**_DMSO_**). The analysis was carried out by applying a quadratic model to MCC-m% dissolution data.

Based on this quadratic model, we constructed the corresponding Pareto charts for the four IL–DMSO BMs [27]. In Figure 5, the bars extending past the vertical (red) line indicate values reaching statistical significance (*p* = 0.05). Thus, both experimental variables were statistically relevant to Cel dissolution; both have comparable relevance, in agreement with previously published data on cellulose dissolution (vide supra).

**Figure 6:**
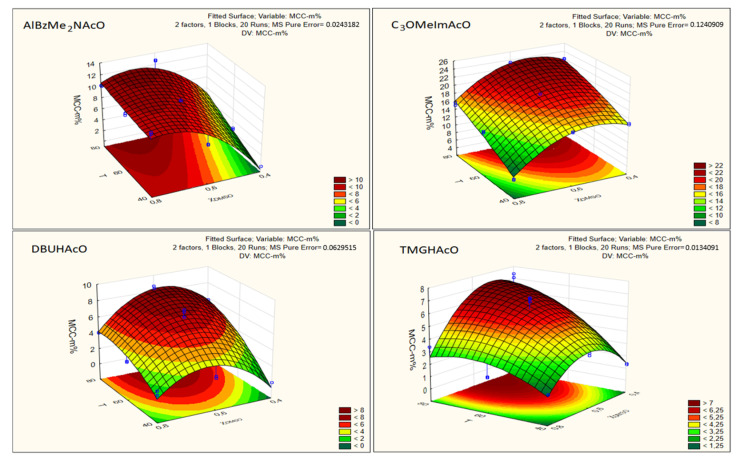
Response–surface plots for the dependence of the mass fraction of dissolved Cel (MCC-m%) on T and (χ**_DMSO_**). The color codes (green → yellow → red → deep red) indicate the increase of MCC-m% as a function of increasing the experimental variable.

We used the data in Table 1 to generate two important pieces of information, namely, the response surfaces [28] and regression equations that correlate cellulose dissolution with the experimental variables. Figure 6 shows the responses of MCC dissolution to T and χ**_DMSO_** for the investigated IL–DMSO BMs. All of the color-coded response surfaces show that Cel dissolution increased as a function of increasing the temperature, and their maximum values (at the same T) were approximately 0.6 (χ**_DMSO_**). The response surfaces of Figure 6 are in agreement with the abovementioned data on the efficiency of Cel dissolution in IL–DMSO BMs, namely, as a function of increasing T. The Cel-m% shows a “bell-shaped” curve (increase → maximum → decrease) as a function of increasing the concentration of DMSO in the BM. This (bell-shaped) behavior is related to competition for the solvation of the AGU hydroxyl groups by ions of the IL versus DMSO. Thus, the initial dilution of the IL with DMSO increased the dissociation of the ionic solvent, leading to efficient IL–Cel interactions (both hydrogen-bonding and hydrophobic interactions) and enhanced biopolymer dissolution. The competition between both solvent components for the hydroxyl groups of the AGU increased, however, on further dilution with DMSO, leading to a decrease in MCC-m%. The reason is that DMSO, unlike the IL, causes swelling but not dissolution of cellulose [29,30,31].

Additionally, we calculated the regression equations, shown in Table 2, that quantify, *for the first time*, this dependence.

Regarding Table 2, we have the following comments:

1. In order to compare the regression coefficients *directly*, the values of T and χ**_DMSO_** were reduced, so that they varied between 0 and 1, before being subjected to the regression analysis;
2. The magnitudes of the regression coefficients indicate the susceptibility/response of the phenomenon studied (i.e., Cel dissolution) to the experimental variables. In addition to expressing the concentration of dissolved Cel as MCC-m%, we also report our data as MCC-χ. The latter scale is more fundamental because differences in the molar masses of mixture components (Cel, CB, IL, co-solvent) do not affect the numerical value of the component. Additionally, the mole fraction scale permits calculation of the number of IL and DMSO molecules required to dissolve Cel. For example, maximum MCC dissolution was observed at 80 °C and χ**_DMSO_** approximately 0.6 (Figure 6). Under these conditions, the molar ratios IL:DMSO per AGU were: AlBzMe_2_NAcO, 3.1:4.7; C_3_OMeImAcO, 1.7:2.5; DBUHAcO, 5.4:8.1, TMGHAcO, 9.7:14.9. Thus, the most efficient solvent system was that based on imidazole because it required smaller numbers of IL and DMSO molecules to dissolve Cel. We recommend that the mole fraction scale should be employed to compare the efficiency of different Cel solvents;
3. As argued above, we concentrated on the regression coefficient of the second (T) and the fourth (χ**_DMSO_**) terms of the equations listed in Table 2. These showed that both variables had comparable effects on MCC dissolution, at least in the range studied, in agreement with Pareto’s chart (Figure 5) and the abovementioned data in the literature where only one variable at a time was changed. Although the two concentration scales (i.e., MCC-m% and MCC-χ) resulted in different values of regression coefficients, the trends were qualitatively similar;
4. The excellent calculated correlation coefficients (*R*^2^) were satisfactory and show the importance of using *dedicated dissolution equipment* and a consistent dissolution protocol, and both ensure reproducible results as argued elsewhere [15];
5. Regarding the aprotic ILs, C_3_OMeImAcO showed a larger decrease of MCC-m% than AlBzMe_2_AcO as a function of increasing χ**_DMSO_**, from 0.6 to 0.8. As discussed elsewhere [21], the oxygen atom of the C_3_*O*- moiety is a Lewis base, capable of forming intramolecular hydrogen bonds with C2-*H* of the imidazolium ring, in addition to intermolecular bonds with the hydroxyl groups of the AGU. It is possible that the efficiency of intermolecular hydrogen bonding is impaired at higher T due to the concomitant large increase of entropy;
6. To test the robustness of these correlations, we calculated MCC-m% under conditions other than those employed to generate the statistical models, and also determined the concentration of dissolved MCC *experimentally*. Table 3 shows the excellent agreement between the predicted and experimental values with the difference ranging between 0.7% and 6.2%. Entry 4 is interesting because the temperature employed was outside the T range investigated.
7. Many authors used solvatochromic parameters to assess the efficiency of Cel solvents. In this regard we note:
  − We used *E**_T_***(WB) because values of λ**_max_** of the corresponding solvatochromic peak showed a much larger dependence on the experimental conditions than other probes employed for calculation of, for example, Lewis basicity (*SB*; S = solvent). Consider the following values of Δλ**_max_** of WB that we observed for CB/TMGHAcO-DMSO on changing χ**_DMSO_** from 0.4 → 0.8: 10 nm (T = 40 °C) and 16.7 nm (T = 60 °C). The corresponding Δλ**_max_** for *N,N*-dimethyl-4-nitroaniline (one of the homomorphic pair employed to calculate *SB*) were 0.1 nm (T = 40 °C) and 0.6 nm (T = 60 °C). In the present case, therefore, WB is much more sensitive to changes in the experimental variables than other solvatochromic probes that are used to calculate specific Cel-solvent interactions. Note that empirical solvent polarity is related to the parameters that describe the specific solute–solvent interactions by Equation (5) [22,23,24,25,26,27,28,29,30,31,32]:
    *E**_T_***(probe) = *E**_T_***(probe)_0_ + a *SA* + b *SB* + d *SD* + p *SP* (5)
    where *SB* is as defined before; *SA*, *SD*, and *SP* refer to Lewis acidity, dipolarity, and polarizability, respectively. That is, the dependence of *E**_T_***(probe) on the experimental variables reflects collectively the dependence (on the same variables) of hydrogen bonding (as given by *SA* and *SB*) and the hydrophobic interactions (related to *SP*). These two mechanisms of Cel–solvent interactions are central to dissolution of the biopolymer [33,34].
  − *E**_T_***(probe) is, however, a *dependent variable*, i.e., its value is determined by T and χ**_DMSO_**. Indeed, correlations of the values of *E**_T_***(WB) listed in the footer of Table 1(^d^) with T at fixed χ**_DMSO_** and with χ**_DMSO_** at fixed T (correlations not listed) show perfect second-order polynomials with *R*^2^ = 1. Therefore, it is possible, *in principle*, to use *E**_T_***(WB) *instead of* T or χ**_DMSO_** in Table 2, because the medium empirical polarity is strongly correlated with the independent variables studied. The relevant point, however, is that there is theoretical and experimental ground for using *E**_T_***(probe) to assess the efficiency of cellulose solvents.

## 4. Conclusions

Optimization of the physical dissolution of cellulose is important for its applications. The approach of one-at-a-time variation of the experimental variables does not guarantee reaching a true dissolution maximum. Equally important, however, it gives no indication about the relative importance (to Cel dissolution) of the experimental variables. That is, chemometrics should be employed *as shown here for the first time*. Using a specially constructed mechanical stirrer and an established dissolution protocol that ensures reproducibility of the results, we investigated the effects of temperature and BM composition on MCC dissolution. Dimethyl sulfoxide was used as a co-solvent for aprotic AlBzMe_2_AcO and C_3_OMeImAcO and protic DBUHAcO and TMGHAcO ILs. Both IL types have potential industrial application in cellulose regeneration/recycling. Using factorial design with repetitions of the central and peripherical points, we carried out 16 experiments for each IL–DMSO mixture and used a quadratic model to fit the MCC dissolution data. The Pareto plots indicated that both experimental variables were important, and the response surface showed that maximum MCC dissolution occurred at 80 °C and a χ**_DMSO_** of approximately 0.6. The robustness of the statistical model was evidenced by the high correlation coefficients and by the small differences between predicted (by the model) and experimentally determined MCC-m%. Thus, use of chemometrics allows *quantification* of the relative importance of the experimental variables. Equally important, however, this use saves time, labor, and material.

## Figures and Tables

**Table 1:** Dependence of the dissolution of microcrystalline cellulose (MCC) in IL/DMSO binary mixtures on the molecular structure of the IL, the temperature, and the mole fraction of DMSO in the starting binary solvent ^a^.

		AlBzMe_2_NAcO		C_3_OMeImAcO		DBUHAcO		TMGHAcO	
Temperature (°C)	χ_DMSO_	MCC-m% ^b^	MCC-χ ^b^	MCC-m% ^b^	MCC-χ ^b^	MCC-m% ^b^	MCC-χ ^b^	MCC-m% ^b^	MCC-χ ^b^
**40**	**0.4**	0.0 ^c,d^	0.0	14.5 ^c,d^	13.7	0.0 ^c,d^	0.0	1.3 ^c,d^	1.1
**40**	**0.4**	0.0 ^c^	0.0 ^c^	14.8	13.8	0.0 ^c^	0.0 ^c^	1.4	1.1
**60**	**0.4**	2.6	2.5	18.0	16.9	3.2	2.9	4.5	3.8
**80**	**0.4**	4.0	4.3	22.0	20.8	5.8	5.4	7.4	6.3
**80**	**0.4**	4.3	4.5	22.2	21.0	5.5	5.0	7.1	6.0
**40**	**0.6**	6.2	5.5	15.3	12.4	2.2	1.7	3.4	2.4
**60**	**0.6**	9.3	8.2	19.9	16.2	7.5	5.9	6.9	5.1
**60**	**0.6**	9.6	8.4	19.0	15.4	7.6	6.1	6.7	4.9
**60**	**0.6**	9.5	8.5	19.9	16.3	7.6	6.1	6.9	5.1
**60**	**0.6**	9.6	8.4	19.0	15.4	7.9	6.2	7.0	5.1
**80**	**0.6**	12.8	11.3	23.5	19.4	8.7	6.9	5.4	3.9
**40**	**0.8**	10.3	7.2	7.0	4.5	1.9	1.2	1.4	0.8
**40**	**0.8**	10.0	7.0	6.7	4.3	1.4	0.9	1.4	0.8
**60**	**0.8**	9.3	6.4	13.0	8.7	2.9	1.8	1.9	1.1
**80**	**0.8**	10.2	7.1	15.0	10.1	4.1	2.7	3.4	2.0
**80**	**0.8**	10.0	7.2	15.8	10.6	4.0	2.6	3.4	2.0

^a^ Abbreviations: AlBzMe_2_NAcO, allylbenzyldimethylammonium acetate; C_3_OMeImAcO, 1-(2-methoxyethyl)-3-methylimidazolium acetate; DBUAHcO, 1,8-diazabicyclo[5.4.0]undec-7-ene-8-ium acetate; TMGAHcO, tetramethylguanidinium acetate; DMSO, dimethyl sulfoxide; IL, ionic liquid; MCC, microcrystalline cellulose. ^b^ MCC-m% is the percentage mass fraction of dissolved cellulose = (cellulose mass/(cellulose mass + mass of (LI+DMSO)) × 100. MCC-χ is the mole fraction of dissolved cellulose, calculated as hydroglucose units = (number of AGU moles/(number of AGU moles + number of IL moles + number of DMSO moles)). Based on the results of the central points for which we had more data points, we calculated the uncertainty from: ((MCC-m%)_maximum_ − (MCC-m%)_minimum_/(MCC-m%)_maximum_ ) × 100. The following are the uncertainties calculated: 3%, AlBzMe_2_NAcO; 4.5%, C_3_OMeImAcO; 5%, DBUHAcO; 4.3%, TMGHAcO. ^c^ In this experiment, MCC did not dissolve *completely after the first biopolymer addition*, after 3 h. ^d^ The following calculated values of the medium empirical polarity (cellobiose + IL + DMSO) and *E**_T_***(WB) are listed in the following order: ionic liquid, temperature, and *E**_T_***(WB) in kcal/mol for χ**_DMSO_** = 0.4, 0.6, and 0.8, respectively. AlBzMe_2_NAcO: 40 °C, 57.2, 56.8, 56.4; 60 °C, 56.4, 56.0, 55.5; 80 °C, 55.1, 54.8, 54.4. C_3_OMeImAcO: 40 °C, 59.9, 59.0, 57.4; 60 °C, 58.2, 57.5, 56.3; 80 °C, 55.2, 54.5, 53.3. DBUHAcO: 40 °C, 57.7, 57.5, 57.2; 60 °C, 56.7, 56.4, 55.8; 80 °C, 55.0, 54.8, 54.5. TMGHAcO: 40 °C, 58.4, 58.2, 57.5; 60 °C, 58.2, 57.8, 57.2; 80 °C, 57.8, 57.3, 56.8.

**Table 2:** Regression equations for the dependence of the concentration of dissolved cellulose on the temperature (T) and the mole fraction of DMSO (χ**_DMSO_**).

**Dependence of MCC-m% on T and χ_DMSO_**			
**Entry**	**Ionic Liquid**		*R* ^2 a^
1	AlBzMe_2_AcO	MCC-m% = −0.59 + 5.23 T + 0.46 T^2^ + 23.63 (χ_DMSO_) − 13.94 (χ_DMSO_)^2^ − 4.20 T × (χ_DMSO_)	0.946
2	C_3_OMeImAcO	MCC-m% = 14.38 + 9.57 T − 2.10 T^2^ + 10.18 (χ_DMSO_) − 17.20 (χ_DMSO_)^2^ + 1.10 T × (χ_DMSO_)	0.986
3	DBUHAcO	MCC-m% = −0.65 + 10.54 T − 4.08 T^2^ + 15.04 (χ_DMSO_) − 13.49 (χ_DMSO_)^2^ − 3.25 T × (χ_DMSO_)	0.936
4	TMGHAcO	MCC-m% = +1.43 + 9.35 T − 4.09 T^2^ + 8.25 (χ_DMSO_) − 8.49 (χ_DMSO_)^2^ − 3.90 T × (χ_DMSO_)	0.883
**Dependence of MCC-χ on T and χ_DMSO_**			
**Entry**	**Ionic Liquid**		*R* ^2 a^
5	AlBzMe_2_AcO	MCC-χ = −0.45 + 5.33 T + 0.30 T^2^ + 21.78 (χ_DMSO_) − 15.05 (χ_DMSO_)^2^ − 4.46 T × (χ_DMSO_)	0.944
6	C_3_OMeImAcO	MCC-χ = 13.51 + 8.39 T − 1.14 T^2^ + 4.36 (χ_DMSO_) − 13.14 (χ_DMSO_)^2^ + 1.20 T × (χ_DMSO_)	0.992
7	DBUHAcO	MCC-χ = −0.43 + 8.48 T − 2.70 T^2^ + 12.23 (χ_DMSO_) − 10.80 (χ_DMSO_)^2^ −4.20 T × (χ_DMSO_)	0.933
8	TMGHAcO	MCC-χ = +1.18 + 7.82 T − 3.29 T^2^ + 5.19 (χ_DMSO_) − 5.69 (χ_DMSO_)^2^ − 3.80 T × (χ_DMSO_)	0.903

^a^*R*^2^ is the regression correlation coefficient.

**Table 3:** Comparison of predicted and experimental cellulose dissolution data ^a^.

Entry	Ionic Liquid	Variable Employed	(WT%) Calculated	(WT%) Experimental	Δwt%
1	AlBzMe_2_NAcO	60 °C/0.70 χ_DMSO_	10.4	10.0	−4.0
2	AlBzMe_2_NAcO	55 °C/0.50 χ_DMSO_	6.1	5.8	−5.1
3	C_3_OMeImAcO	70 °C/0.80 χ_DMSO_	14.2	14.1	−0.7
4	C_3_OMeImAcO	35 °C/0.30 χ_DMSO_	10.5	11.0	4.5
5	DBUHAcO	55 °C/0.70 χ_DMSO_	5.5	5.8	5.2
6	DBUHAcO	70 °C/0.50 χ_DMSO_	7.3	7.5	2.7
7	TMGHAcO	55 °C/0.50 χ_DMSO_	5.5	5.2	−5.8
8	TMGHAcO	80 °C/0.70 χ_DMSO_	5.1	4.8	−6.2

^a^ MCC was used in all experiments. Δwt% = ((Experimental MCC-m% − predicted MCC-m%)/Experimental MCC-m%) × 100.

## Supplementary Materials

The following are available online. Figure S1: ^1^H NMR spectra of the ionic liquids synthesized. All spectra (Varian Inova model YH300 spectrometer; 300 MHz for ^1^H; all ILs were dissolved in CDCl_3_). The spectra are for allylbenzyldimethylammonium acetate (AlBzMe_2_NAcO), A; 1-(2-methoxyethyl)-3-methylimidazolium acetate (C_3_OMeImAcO), B; 1,8-diazabicyclo[5.4.0]undec-7-ene-8-ium acetate (DBUHAcO), C; and tetramethylguanidinium acetate (TMGHAcO), D; Table S1 Experimental factorial planning 3^2^, and experiment repetitions.

## List of Abbreviations and Acronyms

AGU	Anhydroglucose unit
AlBzMe_2_NAcO	Allylbenzyldimethylammonium acetate
BM	Binary mixture of solvents
CB	Cellobiose
Cel	Cellulose
DBUAHcO	1,8-Diazabicyclo[5.4.0]undec-7-ene-8-ium acetate
DMSO	Dimethyl sulfoxide
IL	Ionic liquid
MCC	Microcrystalline cellulose
TMGHAcO	Tetramethylguanidinium acetate
*E_T_*(WB)	Empirical polarity of the medium in kcal/mole, calculated from the UV-Vis spectra of the solvatochromic probe 2,6-dichloro-4-(2,4,6-triphenylpyridinium-1-yl)phenolate (WB)
*SA*	Lewis acidity of the medium
*SB*	Lewis basicity of the medium
*SD*	Medium dipolarity
*SP*	Medium polarizability
C_3_OMeImAcO	1-(2-methoxyethyl)-3-methylimidazolium acetate
