# Supplementary material for: Cellulose Dissolution in Mixtures of Ionic Liquids and Dimethyl Sulfoxide: A Quantitative Assessment of the Relative Importance of Temperature and Composition of the Binary Solvent
Source: Molecules. 2020 Dec 17;25(24):5975. doi: 10.3390/molecules25245975 (PMC7766604; doi:10.3390/molecules25245975)
Supplement: Supplementary file 1 [file molecules-25-05975-s001.pdf]

Supplementary Material for

Cellulose dissolution in mixtures of ionic liquids and dimethyl sulfoxide. Quantitative assessment of the relative importance of the temperature and composition of the binary solvent.

Marcella T. Dignani, Thaís A. Bioni, Thiago R. L. C. Paixão and Omar A. El Seoud,*

Institute of Chemistry, the University of São Paulo, 748 Prof. Lineu Prestes Av., 05508-000 São Paulo, SP, Brazil; marcelladignani2@gmail.com (M.T.D.); tha.bioni@hotmail.com (T.A.B.); trlcp@iq.usp.br (T.R.L.C.P.)

* Correspondence: elseoud.usp@gmail.com

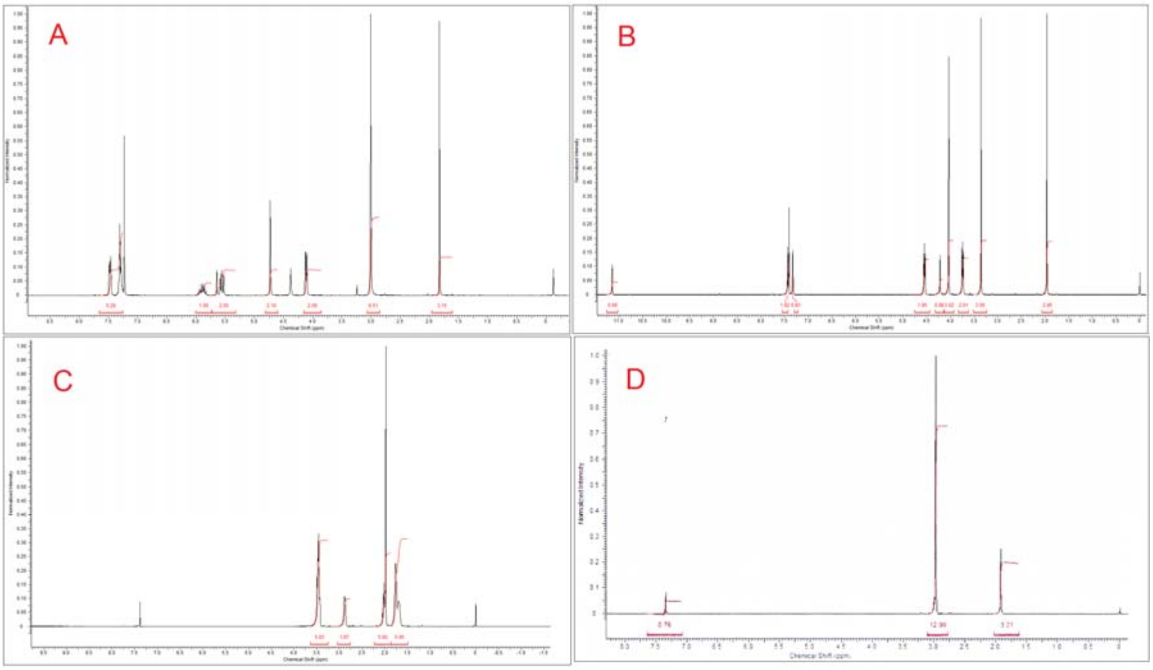

Figure S1. ^{1}H NMR spectra of the ionic liquids synthesized. All spectra (Varian Inova model YH300 spectrometer; 300 MHz for ^{1}H; all ILs were dissolved in $CDCl_3$). The spectra are for allylbenzyldimethylammonium acetate ($AlBzMe_2NAcO$), A; 1-(2-methoxyethyl)-3-methylimidazolium acetate ($C_3OMeImAcO$), B; 1,8-diazabicyclo[5.4.0]undec-7-ene-8-ium acetate (DBUHAcO), C; and tetramethylguanidinium acetate (TMGHAcO), D.

Table S1. Experimental factorial planning 3^2, and experiment repetitions. [a.]

Entry	T, °C	DMSO
**1**	-1	-1
**2**	-1	-1
**3**	0	-1
**4**	1	-1
**5**	1	-1
**6**	-1	0
**7**	0	0
**8**	0	0
**9**	0	0

**10**	0	0
**11**	1	0
**12**	-1	1
**13**	-1	1
**14**	0	1
**15**	1	1
**16**	1	1

See text for definitions of (−1, 0, 1). The randomized order of experiments was generated by the Statistica software, see Experimental for details.
